# The Synergistic Effect of Polyphosphates Acid and Different Compounds of Waste Cooking Oil on Conventional and Rheological Properties of Modified Bitumen

**DOI:** 10.3390/ma15238681

**Published:** 2022-12-05

**Authors:** Wentong Wang, Jin Li, Di Wang, Pengfei Liu, Xinzhou Li

**Affiliations:** 1 School of Highway, Chang’an University, Xi’an 710064, China; 2 School Transportation Civil Engineering, Shandong Jiaotong University, Jinan 250357, China; 3 Department of Civil Engineering, Aalto University, 02150 Espoo, Finland; 4 Hangzhou Telujie Transportation Technology Co., Ltd., Hangzhou 311121, China; 5 Institute of Highway Engineering, RWTH Aachen University, D-52074 Aachen, Germany

**Keywords:** modified bitumen, waste cooking oil, polyphosphate acid, rheological property, conventional property

## Abstract

In order to conserve non-renewable natural resources, waste cooking oil (WCO) in bitumen can help lower CO_2_ emissions and advance the environmental economy. In this study, three different components of WCO were isolated and then, together with polyphosphoric acid (PPA), used separately as bitumen modifiers to determine the suitability of various substances in WCO with PPA. Conventional tests, including penetration, softening point temperature, and ductility, and the dynamic shear rheology (DSR) test, including temperature sweep and frequency sweep, were used to evaluate the influence of WCO/PPA on the traditional performance and rheological properties at high and low temperatures. The results indicate that WCO reduced the ductility and penetration value, when the use of PPA increased the softening point temperature and high-temperature performance. Compared to reference bitumen, the rutting factor and viscous activation energy (Ea) of bitumen modified with 4% WCO and 2% PPA has the most significant increase by 18.6% and 31.5, respectively. All components of WCO have a significant impact on improving the low-temperature performance of PPA-modified bitumen. The performance of the composite-modified bitumen at low temperatures is negatively affected by some waxy compounds in WCO, such as methyl palmitate, which tends to undergo a solid–liquid phase change as the temperature decreases. In conclusion, the inclusion of WCO/PPA in bitumen offers a fresh approach to developing sustainable pavement materials.

## 1. Introduction

Over the past 30 years, the construction of road infrastructure has seen extraordinary progress worldwide [1]. As a result, massive amounts of non-renewable natural resources are frequently utilized to build road infrastructure, including natural aggregates and bitumen derived from fossil fuels [2], which increases the cost of building and garners significant media attention about the environment and natural resources. In the meantime, the rate of global garbage creation is increasing, with annual production reaching over 1.5 billion tons [3]. The government faces tremendous challenges when it comes to handling, storing, and transporting waste [4]. Typically, the debris was handled by piling and burying, which reduced the amount of landfill area and harmed [5]. Finding a novel method of treating garbage is, therefore, urgently needed. One promising solution is to recycle and reuse construction demolition waste, especially recycled asphalt pavement (RAP), in road construction [6]. Lowering CO_2_ emissions, increasing land-use efficiency, protecting resources, and advancing the economy are all beneficial [7]. Nowadays, up to 80% of RAP can be used in bitumen road construction in Europe [8,9]. However, there are still knowledge gaps to be filled, such as whether the sorting of waste resources be efficiently applied to bitumen materials to improve engineering performance.

Household garbage is being produced in significant quantities as a result of the world population’s fast rise. Waste cooking oil (WCO) is one of the most well-known types [10,11], which contains large amounts of polycyclic aromatic hydrocarbons and benzo(a)pyrene, posing a serious threat to human health and the safety of the ecological environment [12]. WCO pollutes the natural results to different degrees because there is no effective way to treat it [13,14]. Statistics reveal that in China alone, there are 7 million tons of WCO produced annually, but only 1 million tons of that is correctly reused because of technical and equipment issues [15]. The efficient utilization of WCO resources has also become a hotly debated issue among academics.

Recently, much attention has been paid to the potential application of WCO in bitumen modification and rejuvenation. Plenty of studies have shown that WCO has been used as an asphalt rejuvenation agent (ARA), modifier, additive for warm mix bitumen, and raw material for the production of bio-based bitumen [14,15,16,17,18]. Chen [19] added 3%, 4%, 5%, 6%, and 7% of waste cooking oil to three different types of aged bitumen and evaluated the regeneration effect of waste cooking oil by rheological performance tests. The results showed that the application of WCO in bitumen led to a decrease in viscosity, which was due to the low molecular weight of WCO, low viscosity, good flowability, and high viscosity reduction and drag reduction effect. Azahar collected WCO at different levels of use and mixed it with aged bitumen to prepare recycled bitumen. The results show that the frequency and manner of WCO use affect the performance of recycled bitumen [20]. Meanwhile, Chen studied the regeneration effect of waste oil on bitumen with the same degree of aging. The results of the study found that the type and content of fatty acids in the waste oil were important factors affecting the regeneration effect [19]. When the aforementioned literature is combined, it is simple to see that the types, sources, and qualities of WCO used in various investigations vary greatly. After varying application techniques and usage frequencies, the same edible oil’s modification effect on bitumen also changes greatly. That is to say, the type and quality of WCO employed are the primary factors that affect how changed bitumen is modified. Additionally, some specific WCO components may potentially harm bitumen’s performance [21]. In view of this, it is important to study the influence of the composition of WCO on bitumen properties and to pre-treat WCO to classify and manage it, thus improving the efficiency of WCO utilization [22,23].

Modifiers and additives were commonly used to improve the bitumen’s rheological properties in high-performance bitumen pavements [24,25,26]. Among them, both physical modification and chemical modification of polyphosphoric acid (PPA) were attempted in previous studies [27]. PPA-modified bitumen is gaining interest due to the more prominent and less costly modification effect. From the analysis of the rheological test results, it was validated that the addition of PPA could benefit the high-temperature performance and rutting deformation resistance of bitumen [28,29]. Moreover, PPA can react with the alcohols in bitumen [30]. The addition of PPA was found to contribute to the increase in asphaltene content. However, as a result of the bitumen’s reaction with PPA, it becomes brittle and less ductile [29,31]. Therefore, the combined use of WCO/PPA additives to modify the low-temperature properties of bitumen could be an option, this may be attributed to the high amount of light component of WCO. The interplay of WCO and PPA not only potentially balances out each other’s flaws, but also offers promising opportunities for long-term use. the effect of the composition of WCO on the performance of modified bitumen is not clear. In summary, the major goal of this work was to examine the impact of various WCO constituents on the rheological characteristics of PPA-modified bitumen at high and low temperatures.

In view of the above, the WCO was firstly, fractionated into three stable fractions: light component (LC), intermediate component (IC), and heavy component (HC). Next, the three components were blended with the matrix bitumen at a 4% admixture rate, respectively. Then, the composite-modified bitumen was prepared by adding PPA (0.5%, 1%, 1.5% and 2%) using high-speed mixing. In order to determine its basic function, the conventional properties, including penetration value, softening point temperatures, and ductility, of the bitumen were evaluated. Finally, the high- and low-temperature rheological properties of WCO/PPA-modified bitumen was evaluated and discussed. Due to the many occurrences of proper nouns in this paper, all abbreviations have been listed in Table 1 for ease of reading.

## 2. Materials and Methods

### 2.1. Raw Materials

#### 2.1.1. Bitumen

SK-90# bitumen was selected as the original bitumen, and the base performance is listed in Table 2. All the parameters fit the requirement of the active Chinese national standard GB JTG E20-2011.

#### 2.1.2. PPA

According to the literature, the commonly used PPA modifier content range from 0.5% to 2.5% by weight (wt%) of the original bitumen [27,29,32,33,34]. Given that PPA prepolymers have a positive impact on bitumen’s high-temperature properties, this study tried to select four low PPA contents, as low as 0.5 wt%, 1 wt%, 1.5 wt%, and 2 wt%. The PPA was provided by Shandong Hui’an Chemical Co., Ltd. (Jinan, Chian), and Table 3 provides a list of PPA’s fundamental characteristics.

#### 2.1.3. WCO

**Figure 1:**
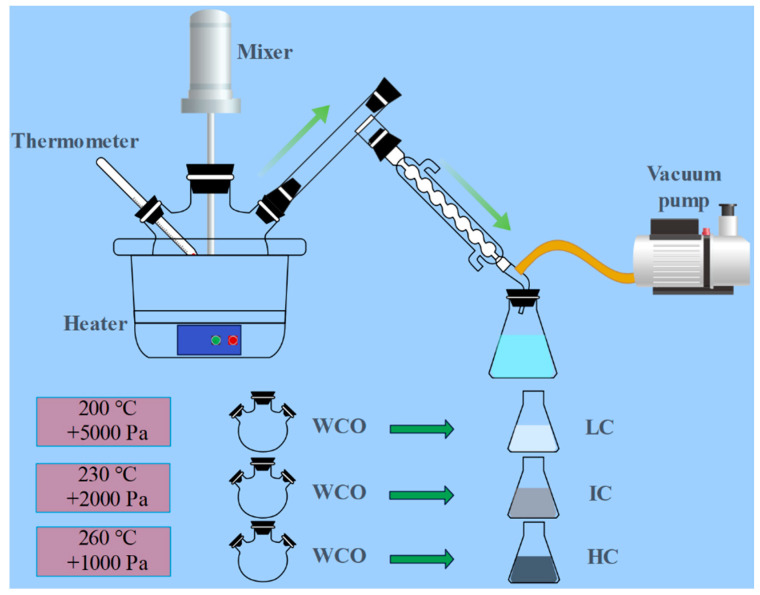
Schematic diagram of laboratory extraction of different compositions of WCO.

The quality and properties of WCO vary greatly from source to source and type to type, which is a major factor in determining the performance of modified bitumen. Therefore, in practical engineering applications, it is important to first ensure that the WCO is sourced consistently. WCO was provided by an oil refinery in Shandong. In this study, WCO was separated by vacuum distillation, and the separation process is shown in Figure 1. The collected LC, IC, and HC processes are shown in Figure 1. LC, IC, and HC were internally doped at 4 wt%. Table 4 lists the characteristics of the three components that were produced by vacuum distilling WCO. Some of the basic performance parameters are presented in Table 4.

### 2.2. Preparation of Samples

**Figure 2:**
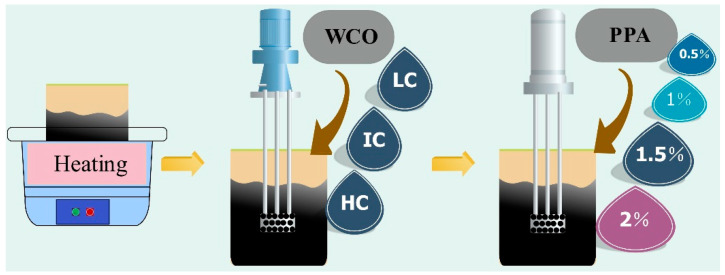
The preparation process of WCO\PPA-modified bitumen.

A high-speed shear mixer was used to prepare the WCO/PPA-modified bitumen, and the procedure is shown in Figure 2. Firstly, the original bitumen was heated in the oven at the temperature of 135 °C. The 4% of LC, IC, and HC were added to the bitumen and mixed for 10 min at 3500 rpm to create WCO-modified bitumen. Then, 0.5%, 1%, 1.5%, and 2% of PPA were added to bitumen. After stirring for 20 min with 4000 r/min continuously, 12 kinds of bitumen modified by WCO/PPA are obtained. The WCO/PPA-modified bitumen combination and abbreviation are shown in Figure 2.

### 2.3. Tests Methods

#### 2.3.1. Conventional Tests

The 25 °C penetration, softening point temperatures, and 5 °C ductility of modified bitumen were carried out by following the active Chinese standards. Three replicates were used in these experiments.

#### 2.3.2. Brookfield Viscosity Test

The temperatures used for the Brookfield viscosity tests were 120 °C, 135 °C, 150 °C, and 165 °C. The whole collection of bitumen shown in Figure 2 was examined in three samples. The less viscous the bitumen is at a high temperature, the more accessible construction is generally.

Generally speaking, the viscous activation energy (Ea) can be used as a gauge to assess how temperature affects various types of materials. As the temperature rises, the thermal motion of molecules intensifies and the spacing between them widens. Ea is the amount of power needed to transport a deformation unit from its beginning location to a nearby “hole”. As Ea increased, the temperature sensitivity decreased. The following are possible ways to determine Ea using the Arrhenius equation [35]:

(1) $$\mu \left(T\right)=B\cdot e^{\frac{E_{a}}{RT}}$$

(2) $$\ln\left(\mu \left(T\right)\right)=\ln\left(B\right)+\frac{E_{a}}{RT}$$

where μ is viscosity (Pa⋅s); B represents the regression coefficient; R represents the universal gas constant; T represents the absolute temperature.

### 2.4. Rheological Properties Tests

In order to produce the rheological indicator, which can be used to research the high-temperature performance and viscoelasticity of bitumen, the modified bitumen was subjected to the temperature sweep (TS) test at a temperature range of 58 °C to 82 °C with an increment of 6 °C. To determine the viscoelastic property of bitumen at 40 °C with a frequency range of 0.1 rad/s to 100 rad/s, the frequency sweep (FS) test was performed.

The maximum strain of the bitumen is a combination of recoverable and non-recoverable deformation. In order to obtain better rutting resistance, the value of non-recoverable deformation should be lower. It is known from the variational form of the Shenoy equation [36], as in Equation (3), when the Shenoy parameter (G*/(1 − (1/TanδSinδ))) should be maximized.

(3) $$\%\gamma_{ur}=\frac{100\sigma }{G^{*}/\left(1-\frac{1}{Tan\delta sin\delta }\right)}$$

where γ_ur_ is the unrecovered strain, σ is the applied stress level, G* is the complex modulus, δ is the phase angle.

### 2.5. Bending Beam Rheometer (BBR) Test

The low-temperature characteristics of modified bitumen were assessed by using the BBR test and −12 °C and −18 °C were chosen as the trial temperatures. The evaluation indices used were the creep rate (m) and stiffness modulus (S) at 60 s of test operation.

## 3. Results and Discussion

### 3.1. Physical Properties

**Figure 3:**
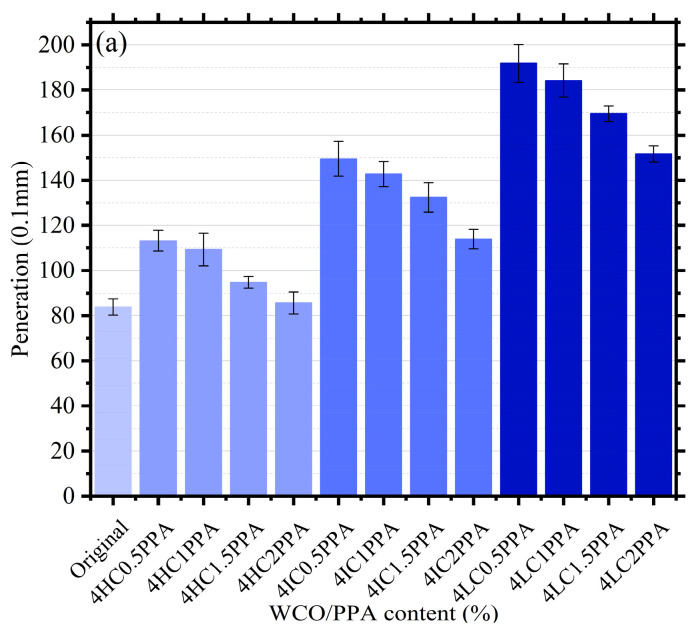

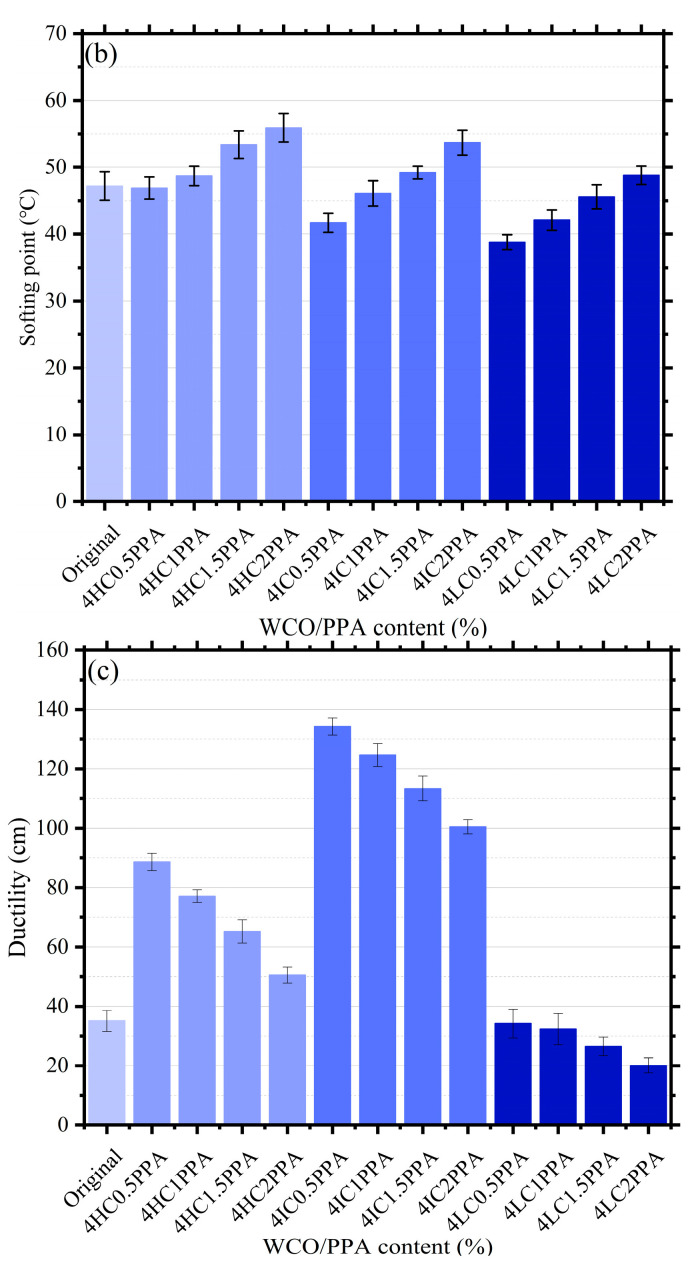
The results of conventional performance: (**a**) Penetration; (**b**) Softening point; (**c**) Ductility.

Figure 3 displays the bitumen’s physical characteristics. All modified bitumen exhibited greater penetrations than original bitumen, as shown in Figure 3a, increasing by 35.1%, 30.4%, 13%, 2.1%, 78.4%, 70.4%, 57.9%, 35.9%, 128.9%, 119.8%, 102.2%, and 81.1%, respectively. As PPA rose, bitumen penetrations decreased. This suggested that the addition of PPA to bitumen increases its hardness, consistency, and ability to resist shear failure, but when the molecular weight of the WCO component drops, the penetration of modified bitumen increases noticeably. It is possible that the light component of WCO causes bitumen to become diluted and softer, which is the cause of this phenomenon [37]. This conclusion is further supported by the finding that 4LC0.5PPA-modified bitumen had the highest penetration.

Figure 3b illustrates a range of bitumen softening points with various WCO/PPA percentages. Compared to penetration, the degree of change was not remarkable. In comparison to the original bitumen, the modified bitumen’s softening points (4LC0.5PPA and 4IC1PPA) were comparable. With decreases of 17.8% and increases of 18.4% in comparison to the original bitumen, the minimum and maximum values of softening points for additives containing 4LC0.5PPA and 4HC2PPA, respectively, were displayed. Generally speaking, with the same constituent concentration of WCO, the PPA content causes a rise in the softening point of modified bitumen. The higher the content of PPA, the greater the influence degree. According to previous studies, PPA can transform bitumen from a sol-gel to a sol-gel structure, this improvement can be attributed to this chemical reaction between bitumen and PPA [38,39].

As a crucial criterion to assess the elastic qualities of bitumen, ductility may be used. The plasticity of bitumen is better the higher the ductility. Figure 3c shows that the addition of WCO/PPA had a greater effect on ductility than on softening point and penetration. As the PPA concentration rose, the ductility decreased when the proportion of WCO components remained constant. This might be the result of the bitumen’s molecular structure becoming more complicated as a result of the reaction between PPA and bitumen, which restricts bitumen molecule movement. The bitumen’s ductility decreases as a result. This showed that the plastic content of WCO-modified bitumen decreases when PPA is present. Despite this, it is clear that modified bitumen has more ductility than original bitumen, increasing by 152.4%, 119.6%, 85.8%, 43.8%, 282.6%, 254.9%, 223.1%, 186.3%, −2.6%, −7.9%, −24.5%, and −42.7%, respectively. Except for LC-modified bitumen, most modified bitumen demonstrated a higher ductility than unmodified bitumen. In particular, for the same PPA content, the IC-modified bitumen has the highest ductility when compared to other samples. One explanation is that the addition of lightweight components alters how bitumen segments move and the system’s free volume, improving the modified bitumen’s resistance to deformation at low temperatures [25,40]. Meanwhile, the high content of light components in LC gives it excellent softening ability at high temperatures, but at low temperatures, it cures as easily as wax and may even destroy the properties of bitumen [41].

### 3.2. Brookfield Viscosity

**Figure 4:**
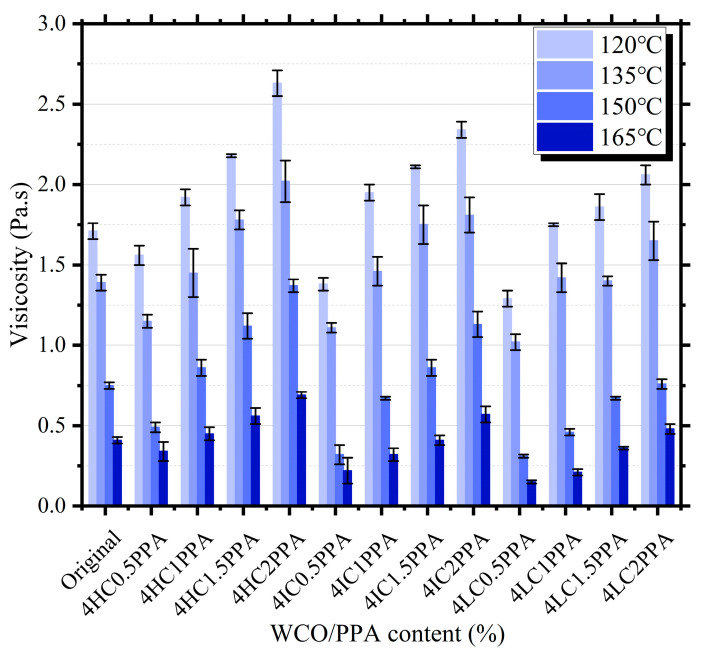
The Brookfield viscosity of the bitumen.

The results of the Brookfield viscosity are represented in Figure 4, which is employed to assess the viscosity–temperature properties. With rising temperatures, it has been discovered that the viscosity of all bitumen kinds decreases. At various temperatures, modified bitumen containing 4HC2PPA had viscosities that were higher than that of the original bitumen by 53.8%, 87.5%, 82.6%, and 68.3%, respectively. The viscosity of 4LC0.5PPA was lower than that of the original bitumen at different temperatures by 24, 26, 6, 58, and 50.1%, respectively. The bitumen’s resistance to shear deformation brought on by external pressures is strengthened by the presence of PPA, as shown by the fact that the viscosity of the bitumen increases with the addition of PPA when the WCO component is kept constant. This is because PPA causes the bi-colloidal tumen’s structure to transition from a sol-gel type to a gel type.

**Figure 5:**
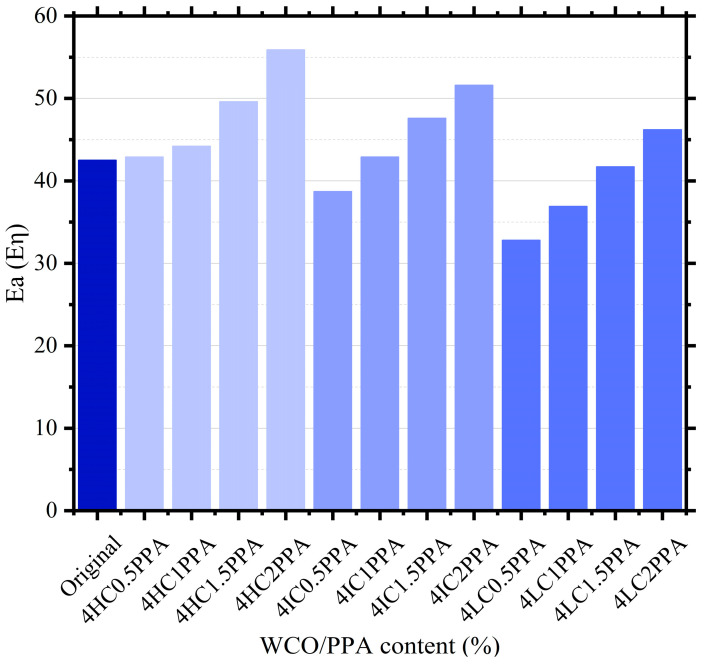
The Ea results of bitumen.

Figure 5 depicts the Ea results of different bitumen. It is clear that the Ea of the original bitumen is affected differently depending on the WCO/PPA ratio. The Ea of 4LC0.5PPA, 4LC1PPA, 4LC1.5PPA, and 4IC0.5PPA were all lower than the original bitumen by 9.7 kJ/mol, 5.6 kJ/mol, 0.8 kJ/mol, and 3.8 kJ/mol, respectively. The Ea of 4HC2PPA, on the other hand, significantly increased and increased to 31.5% in comparison to the original bitumen. As the WCO component reduced for the same proportion of PPA, the Ea of modified bitumen decreased. This could be explained by the bitumen being softened by the light component of WCO, which results in a reduction in the bitumen molecule’s internal restriction.

The ASTM Ai and viscosity–temperature susceptibility (VTSi) for original and WCO/PPA-modified bitumen are summarized in Table 5. The bitumen is often more sensitive to temperature the smaller the absolute value of VTSi. The table shows that Ai and VTSi vary depending on the type and admixture of the modifier, demonstrating how significantly the modifier’s type and admixture affect temperature sensitivity. The findings demonstrate that, when the WCO doping is held constant, the values of parameters Ai and |VTSi| drop as the quantity of PPA increases. However, when PPA is held constant, the WCO admixture raises the values of Ai and |VTSi|. This shows that PPA enhances bitumen’s anti-sensitivity performance whereas WCO enhances bitumen’s temperature sensitivity. Additionally, the LC-modified bitumen has a lower temperature sensitivity than the original bitumen, which is consistent with Ea’s results. The movement of bitumen segments and the system’s free volume are both impacted by the addition of WCO, which could be the cause.

### 3.3. Rheological Properties

#### 3.3.1. Temperature Sweep

**Figure 6:**
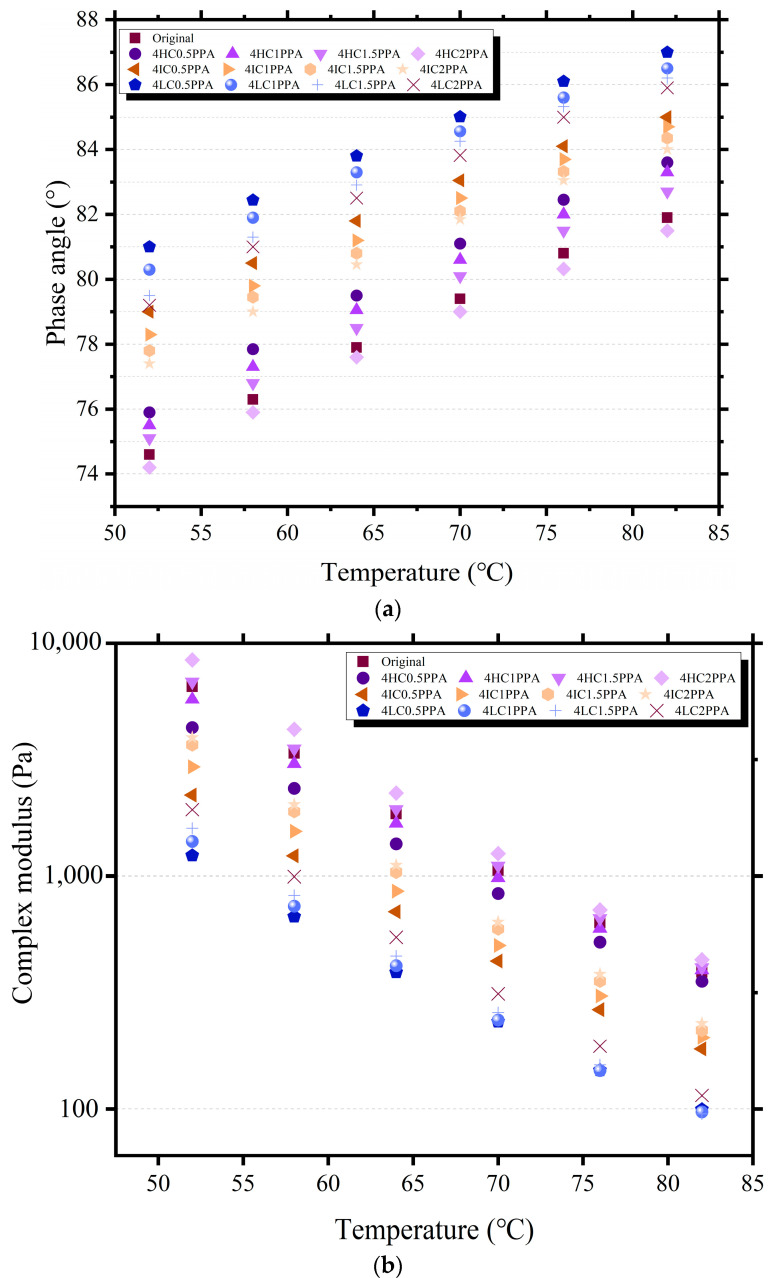

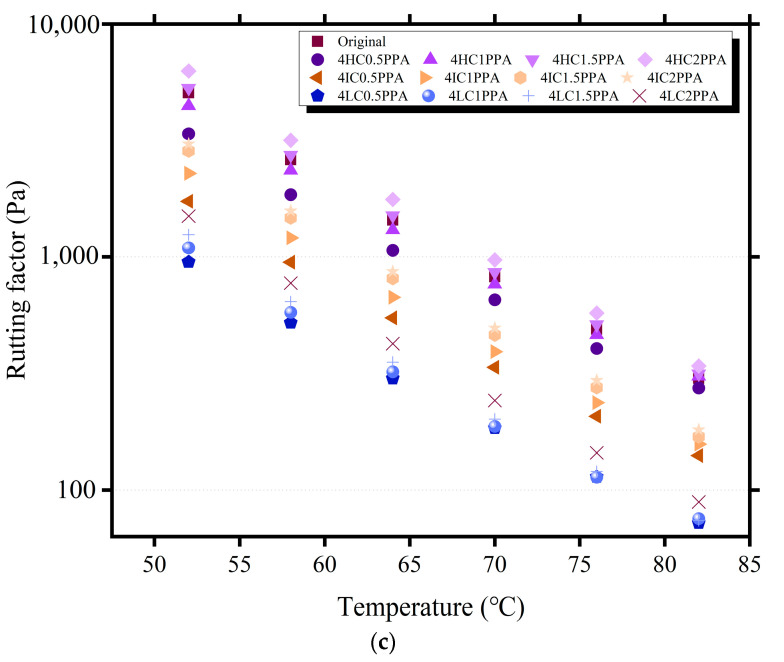
The temperature sweep results of modified bitumen. (**a**) Phase angle of modified bitumen. (**b**) Complex module of modified bitumen. (**c**) Rutting factor of modified bitumen.

As can be seen in Figure 6a, all bitumen’s phase angles were discovered to rise with temperature. That elasticity is declining is demonstrated by this. In particular, three different modifier triads (4HC2PPA, 4LC1PPA, and 4LC2PPA) were shown as an illustration. The phase angles of every modified bitumen were greater than those of the original bitumen, with the exception of 4HC2PPA. Phase angles for the modified 4HC2PPA bitumen were, respectively, 0.49%, 0.47%, 0.36%, 0.45%, 0.64%, and 0.42% smaller than those for the original bitumen within the studied temperature range. The phase angles of the 4LC and 2PPA-containing additives, however, were higher than those of the base bitumen, measuring 8.1%, 7.0%, 6.3%, 6.1%, 5.4%, and 5.0%, respectively. The phase angles for 4LC1PPA have increased at different temperatures by 6.5%, 5.8%, 5.5%, 5.1%, 4.7%, and 4.4% in comparison to those of the original bitumen. It was discovered that the elasticity of modified bitumen increased as the levels of WCO components rose at the same PPA content. When the WCO components are held constant, it is clear that the modified bitumen’s phase angles decreased as the PPA concentrations increased. This phenomenon can be explained by the fact that the addition of PPA causes a change in the bitumen’s sol-gel composition, which prevents WCO from softening the bitumen.

Figure 6b shows the bitumen treated with WCO/PPA complex modulus. It is clear that the modifier’s impact on the original bitumen was different. For instance, the 4LC0.5PPA’s complex modulus has reductions of 58.4%, 59.7%, 58.1%, 55.9%, 53.8%, and 52.7%, respectively, compared to the original bitumen. The complex modulus of 4HC2PPA has increased by around 20.08%, 18.5%, 16.9%, 14.7%, 14.1%, and 10.7%, respectively, when compared to those of original bitumen. The complex modulus of 4LC2PPA is 37.1%, 34.8%, 32.5%, 32.3%, 31.6%, and 28.6% less than that of the original bitumen over the investigated temperature range. The complex modulus of composite-modified bitumen with a higher PPA content and higher WCO molecular weight is, hence, higher.

The rutting factors (G*/Sinδ) at different temperatures are shown in Figure 6c, and they can be used to evaluate the rutting resistance of bitumen. The findings show that a rise in temperature results in a fall in the G*/Sinδ. The G*/Sinδ of modified bitumen increases with an increase in PPA when the WCO component is held constant, demonstrating the PPA’s beneficial influence on high-temperature deformation resistance. In contrast, when the amount of WCO components decreases, the rutting resistance of composite-modified bitumen decreases. When compared to original bitumen at 70 °C, the G*/Sinδ of WCO/PPA-modified bitumen decreased by 9.8%, 4.6%, −5.9%, −18.2%, 17.3%, 14.8%, 12.7%, 12.2%, 23.1%, and 22.4%, respectively. The fact that 4HC2PPA’s G*/Sinδ was 19.1% higher than that of the original bitumens shows that PPA’s incorporation has fixed the original bitumen’s low performance at high temperatures. At high temperatures, however, the difference between WCO/PPA-modified bitumen and unmodified bitumen is the smallest.

Shenoy parameters for the temperature ranges under test are also shown in Table 6. The Shenoy parameter increases dramatically when PPA content is raised to 2%. For instance, when keeping the WCO content constant, the control bitumen Shenoy parameter is 2.9137 kPa, which increased to 3.1244 kPa and 3.8137 kPa, respectively, at 64 °C with the addition of 1.5% and 2% PPA. Such a response, once more, demonstrates that the addition of PPA up to 2% may be beneficial in enhancing rutting performance.

At various temperatures, the Shenoy parameter clearly has a greater value. Additionally, when the PPA content rose, the discrepancy between the Shenoy parameter and the matching G*/Sinδ increased. For example, when the main component of WCO is IC, the difference in Shenoy parameter and G*/Sinδ is 1.3742 kPa for control bitumen, and it rises to 1.6125 kPa, 1.9143 kPa, 2.29111 kPa, and 3.0182 kPa with the addition of 0.5%, 1%, 1.5%, and 2% PPA, respectively, at 58 °C. A possible explanation for this response is the bitumen’s better elastic response brought on by the addition of PPA, which helped to lower the time-dependent unrecovered strain value. This means that in defining the non-recoverable strain value, the Shenoy parameter for evaluating rutting performance is relatively more sensitive than the Superpave rutting parameter to change in WCO/PPA composite-modified bitumen.

**Figure 7:**
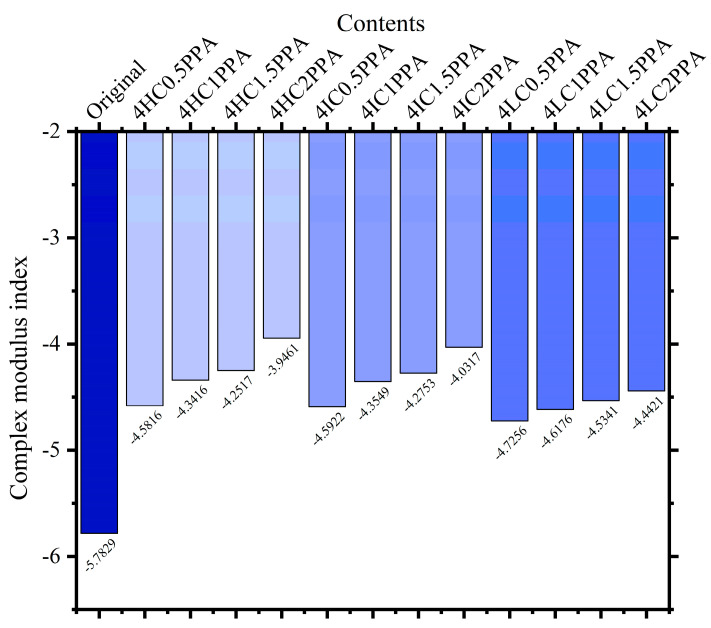
The complex modulus index of different types of bitumen.

Based on the complex modulus at various temperatures, the complex modulus index (GTS) parameter is used to evaluate temperature sensitivity which can be calculated using Equation (4). In general, the greater the absolute value of GTS, the more temperature sensitivity bitumen is. As present, in Figure 7, it is obvious to see that all modified bitumen have lower GTS values than the original bitumen. This suggests that WCO/PPA positively affects the thermal stability of the original bitumen. At the same time, under the same WCO components, the GTS of modified bitumen decreases with the increase in PPA, demonstrating an improvement in bitumen’s temperature stability.

(4) $$GTS=\frac{lgG_{*}-C}{lgT}$$

where: T represents the test temperature; C represents constant.

Typically, the G*/Sinδ of 1 kPa is chosen as the temperature damage threshold for the original bitumen. As can be observed in Figure 6c, all bitumens failed at temperatures lower than 1 kPa at 70 °C, with the exception of 4HC1.5PPA and 4HC2PPA, which had failure temperatures greater than the original bitumen. The failure temperature grows as PPA rises while the WCO component stays constant. This displays how bitumen’s resistance to deformation at high temperatures is improved by the addition of PPA. It is worth noting that the high-temperature rutting resistance of LC-modified bitumen is poor at the same PPA concentration, which is due to the excessive softening phenomenon of bitumen caused by the high number of lighter components in the LC. In contrast, under the conditions of this study, HC can effectively soften the bitumen without compromising its resistance to deformation, Therefore, in engineering applications, the use of this type of component in WCO should be considered first.

#### 3.3.2. Frequency Sweep

**Figure 8:**
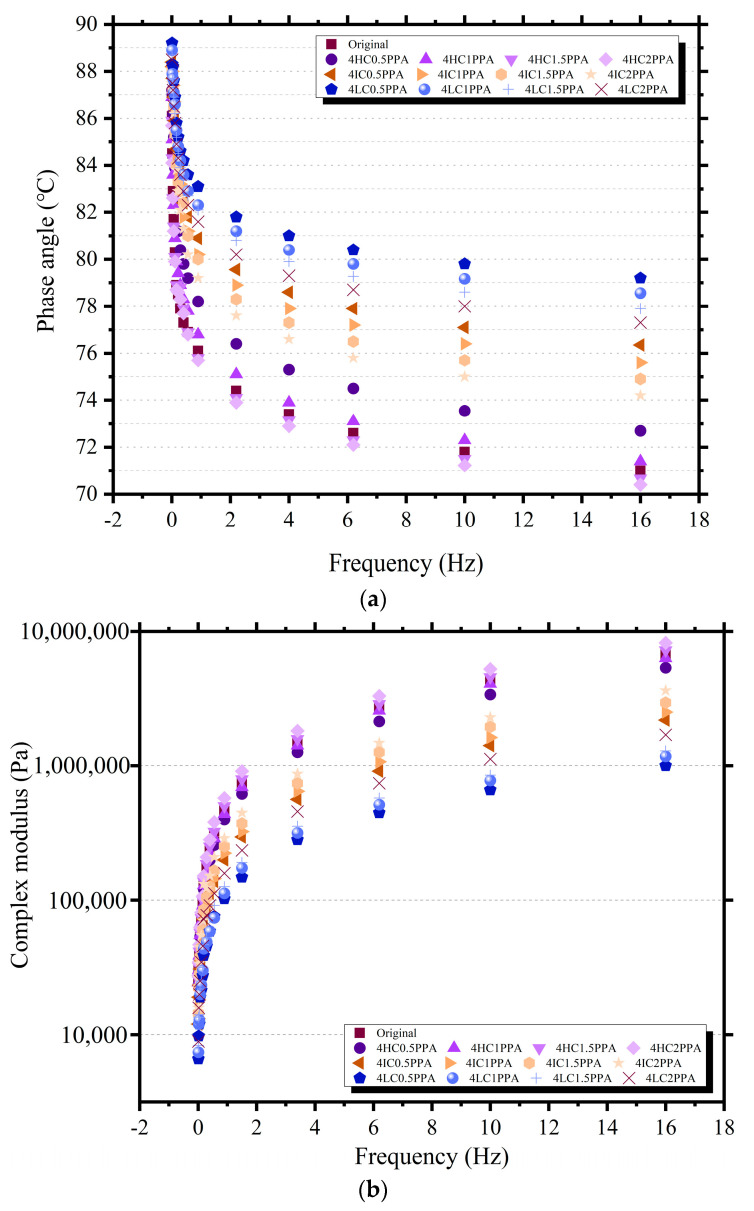

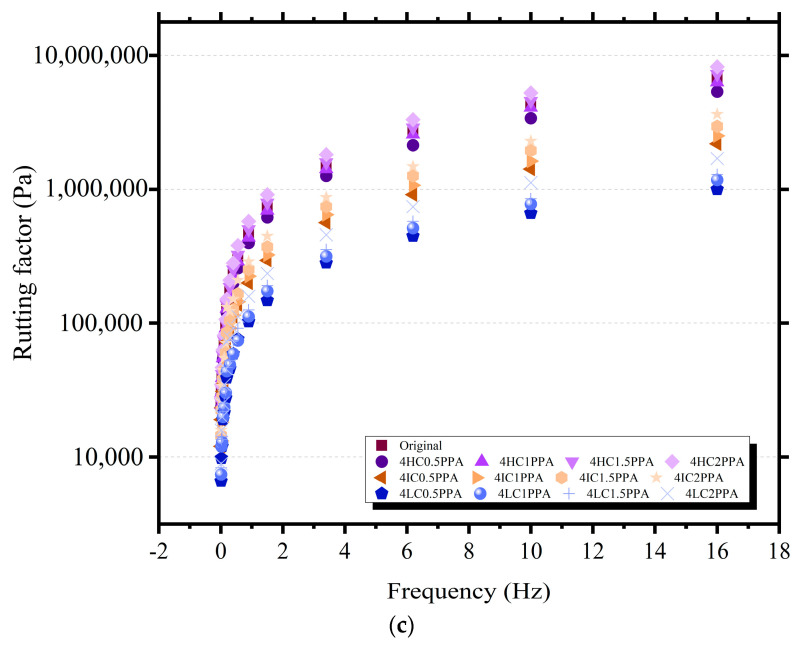
The frequency sweep results of modified bitumen. (**a**) Phase angle of modified bitumen. (**b**) Complex module of modified bitumen. (**c**) Rutting factor of modified bitumen.

Regardless of the ratio of WCO/PPA, the complex modulus rose and the phase angles decreased as frequency increases, as shown in Figure 8. Meanwhile, the higher proportion of PPA can contribute to increase complex modulus and decrease phase angle, and this tendency becomes more obvious with the decrease in the WCO component. It has been established that WCO had a detrimental impact on bitumen’s high-temperature performance [17]. Attribution to the WCO’s softening action, the fluid characteristics of bitumen are improved. However, components with different molecular weights isolated from WCO have different modification effects on bitumen. Specifically, the molecular weight of the substance is inversely proportional to the softening effect on bitumen, due to the fact that the molecular weight determines the mobility of the substance. Therefore, under the combination of the maximum molecular weight of WCO and the maximum content of PPA, the modified bitumen has the best deformation resistance, which is caused by the synergistic effect of the two modifiers. However, the average molecular weight and dispersion coefficient are raised by the addition of PPA, which have a negative impact on the fluidity of bitumen.

### 3.4. BBR

**Figure 9:**
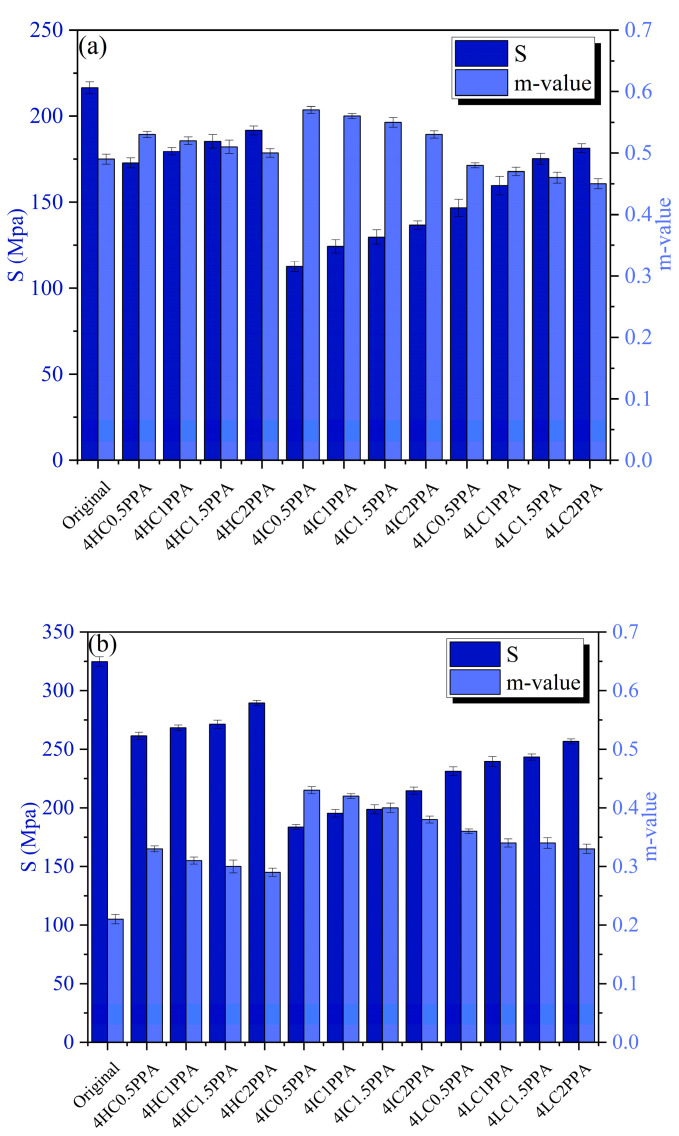
The S and m-value results of modified bitumen: (**a**) −12℃ (**b**) −18℃.

The results of all samples at test temperatures of −12 °C and −18 °C are displayed in Figure 9. It can be shown that when the temperature drops, the S-value rises and the m-value falls. Moreover, it was found that the S of the modified bitumen increased and the m-value decreased as the PPA content increased, indicating that PPA increases the risk of bitumen cracking at low temperatures. The various WCO components, fortunately, improved the cracking resistance of PPA-modified bitumen. Impressively, the incorporation of IC and HC components isolated from WCO improved the cracking resistance of PPA-modified bitumen, except for the low molecular weight IC which weakened the low-temperature cracking performance of the modified bitumen. The crack resistance of IC-modified PPA bitumen is better than the degree of LC and HC modification. This phenomenon can be explained by the difference in molecular weight and composition of substances: (1) The capacity to increase bitumen’s low-temperature qualities is limited by the fact that the heave component is a mixture of several chemicals with the longest carbon chains in WCO; (2) LC, although it has a good softening ability at high temperatures, tends to solidify like wax at low temperatures and can even destroy the high-content properties of bitumen [42]. Therefore, considering its wax-like properties, LC is not recommended to be used as a regenerating agent for RAP.

## 4. Conclusions

This paper aims to investigate the synergistic effect of WCO and PPA on the properties of bitumen. The appropriate WCO/PPA proportion in bitumen was obtained according to the conventional and rheological properties. The following conclusions can be drawn:

The presence of PPA increases the WCO-modified bitumen’s softening point while decreasing its penetration and ductility. PPA can mitigate the impact of WCO’s detrimental effects on high-temperature performance. The penetration and softening point with 4LC0.5PPA and 4HC2PPA present extreme value, rising by 128.9%, −17.8% and 2.1%, 18.4%, the ductility with 4IC0.5PPA and 4LC2PPA present extreme value, rising by 282.6% and −42.7%.

When the WCO content remains constant, the temperature stability of bitumen evaluated by ASTM Ai and VTSi and Ea are enhanced with the raise of PPA proportion. The existence of PPA is beneficial to resist high-temperature deformation.

The results of DSR experiments indicate that PPA can enhance the high-temperature rheology of WCO-modified bitumen. The rutting factors of the 4HC2PPA were 19.1% better than those of the original bitumen, which shows the same result of G*/(1 − (1/TanδSinδ)).

The ability of modified bitumen to perform at low temperatures has no significant linear correlation with the molecular weight of the three WCO components. Since IC is too sensitive to temperature and prone to phase change, it will adversely affect the low-temperature performance of bitumen.

## Figures and Tables

**Table 1:** The abbreviations of proper nouns.

Abbreviations		Abbreviations	
4LC0.5PPA	4%LC + 0.5%PPA	WCO	waste cooking oil
4LC1PPA	4%LC + 1PPA	PPA	polyphosphoric acid
4LC1.5PPA	4%LC + 1.5%PPA	DSR	dynamic shear rheology
4LC2PPA	4%LC + 2%PPA	Ea	viscous activation energy
4IC0.5PPA	4%IC + 0.5%PPA	ARA	asphalt rejuvenation agent
4IC1PPA	4%IC + 1PPA	LC	light component
4IC1.5PPA	4%IC + 1.5%PPA	IC	intermediate component
4IC2PPA	4%IC + 2%PPA	HC	heavy component
4HC0.5PPA	4%HC + 0.5%PPA	TS	temperature sweep
4HC1PPA	4%HC + 1PPA	FS	frequency sweep
4HC1.5PPA	4%HC + 1.5%PPA	G*/(1-(1/TanδSinδ))	Shenoy parameter
4HC2PPA	4%HC + 2%PPA	BBR	Bending beam rheometer
G*/Sinδ	Rutting factor	GTS	grade temperature sensitivity

**Table 2:** Properties of SK-90#.

Technical Indexes	Unit	Results	Test Method
Ductility (5 °C, 5 cm/min)	cm	37.1	T0605
Penetration (25 °C, 100 g, 5 s)	0.1 mm	82.9	T0604
Softening Point	℃	47.3	T0604
RTFOT			
Weight loss	%	−0.066	T0601
Residual penetration ratio	%	57.9	T0604
Residual ductility (5 °C)	cm	21.6	T0605

**Table 3:** The technical indexes of PPA.

Index	Unit	Test Results
Density (25 °C)	g/cm^3^	2.15
Viscosity (85 °C)	mPa.s	562
Iron content	%	≤0.01
Sulfate	%	0.01
P_2_O_5_	%	82.95

**Table 4:** Basic performance properties of WCO.

Index	Unit	Test Results		
		LC	IC	HC
Viscosity (50 °C)	cP	66	85	219
Flash point	°C	197	219	242
Fire point	°C	216	233	264
Density	g/cm^3^	0.89	0.92	0.97
Mechanical impurity	%	0.425	0.002	0
Viscosity (50 °C)	cP	66	85	219

**Table 5:** ASTM Ai-VTSi values for original and modified bitumen.

	Original	4HC0.5PPA	4HC1PPA	4HC1.5PPA	4HC2PPA	4IC0.5PPA	4IC1PPA
Ai	9.251	9.279	9.263	9.011	7.935	9.418	9.284
|VTSi|	3.319	3.367	3.325	3.281	3.243	3.338	3.296
	4IC1.5PPA	4IC2PPA	4LC0.5PPA	4LC1PPA	4LC1.5PPA	4LC2PPA	
Ai	9.025	8.329	10.019	9.846	9.355	8.367	
|VTSi|	3.269	3.271	3.741	3.709	3.328	3.443	

**Table 6:** Average Shenoy parameter values.

	Original	4HC0. 5PPA	4HC1PPA	4HC1. 5PPA	4HC2PPA	4IC0. 5PPA	4IC1PPA	4IC1. 5PPA	4IC2PPA	4LC0. 5PPA	4LC1PPA	4LC1. 5PPA	4LC2PPA
52	9.1742	7.425	8.6143	9.3766	10.1087	4.5115	5.7138	6.6786	6.9724	3.1075	3.1611	3.2238	3.3047
58	5.8879	4.3825	5.4347	6.0867	6.7212	2.6934	2.7951	3.3827	3.6849	2.4372	2.4778	2.5251	2.6064
64	2.9134	2.3851	2.5038	3.1244	3.8137	2.0815	2.1751	2.2575	2.2861	1.8258	1.8592	1.8947	1.9763
70	1.7021	1.6027	1.6655	1.7203	1.7727	1.3119	1.3787	1.4503	1.4803	1.0485	1.0587	1.0902	1.1702
76	1.2156	1.1326	1.1913	1.2356	1.2846	0.8426	0.9013	0.9656	0.9956	0.5826	0.5813	0.7051	0.6856
82	0.8666	0.8264	0.8753	0.8866	0.9197	0.5362	0.5853	0.6166	0.6466	0.4738	0.4757	0.4743	0.4823

## Data Availability

Not applicable.
